# Supplementary material for: Ultrafast structural rearrangement dynamics induced by the photodetachment of phenoxide in aqueous solution
Source: Nat Commun. 2019 Jul 3;10:2944. doi: 10.1038/s41467-019-10989-1 (PMC6610110; doi:10.1038/s41467-019-10989-1)
Supplement: Supplementary file 1 — Supplementary Information [file 41467_2019_10989_MOESM1_ESM.pdf]

Supplementary Information

Ultrafast structural rearrangement dynamics induced by the photodetachment of phenoxide in aqueous solution

Debnath et al.

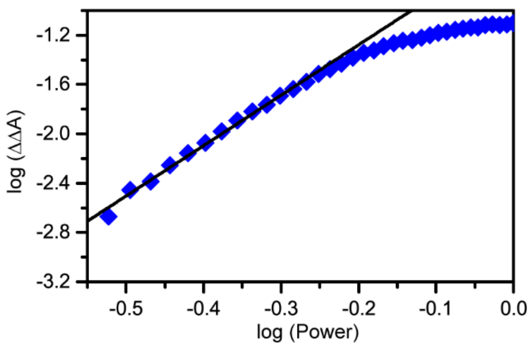

Supplementary Figure 1 | Photon-order measurement for strong-field photodetachment. Log-log plot of $\Delta\Delta A$ vs. power after subtraction of the response from pure liquid water. The slope in the pre-saturation regime is 4.1 ± 0.1.

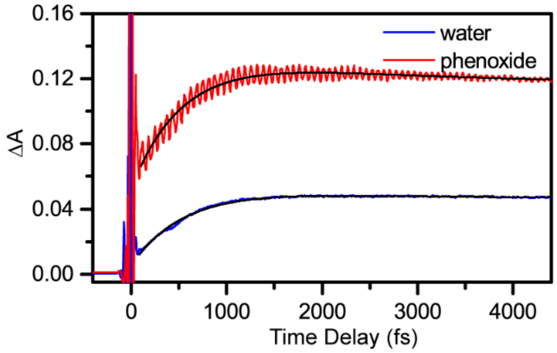

Supplementary Figure 2 | Time trace obtained from ionized pure liquid water. Comparison of time traces recorded at 406 nm obtained from the strong-field ionization of pure liquid water and the photodetachment of phenoxide, along with the fitted lines (black lines). The rising component of both time traces have very similar time constants (0.57 ± 0.01 ps for water and 0.54 ± 0.01 ps for phenoxide), suggesting that they have the same origin.

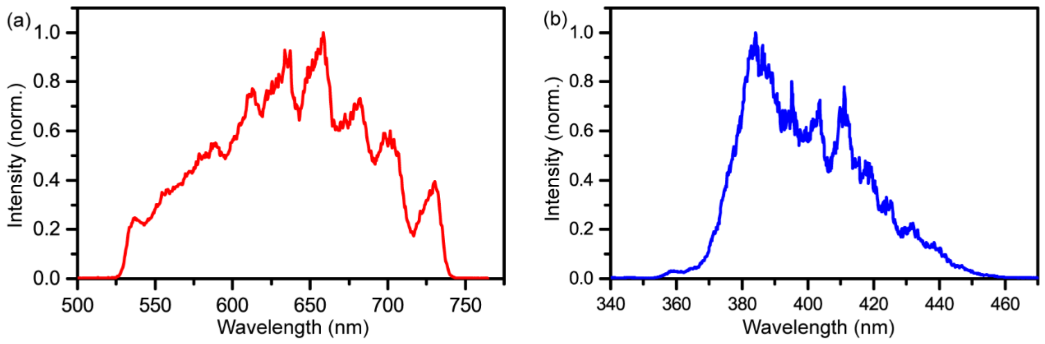

Supplementary Figure 3 | Laser spectra. (a) Spectrum of few-cycle laser pulse used to induce strong-field photodetachment of phenoxide in aqueous solution. (b) Spectrum of probe pulse.

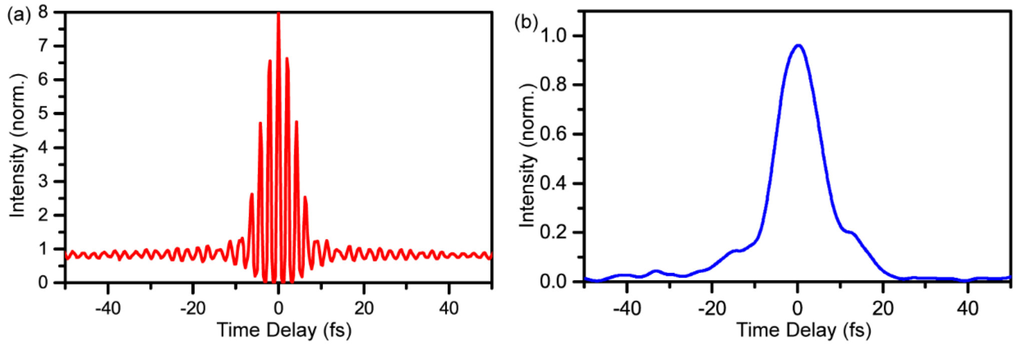

Supplementary Figure 4 | Auto- and cross-correlation traces. (a) Second-order interferometric autocorrelation trace of the few-cycle laser pulse used for the strong-field photodetachment of phenoxide in aqueous solution reveals a pulse duration of 6 fs FWHM. (b) The pump-probe cross-correlation, measured via difference frequency mixing, reveals a FWHM of 12 fs. Note that the actual instrument response is expected to be narrower (~11 fs) because of the multiphoton nature of the strong-field photodetachment process (see below).

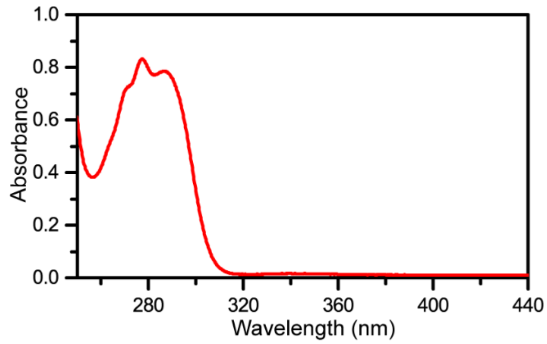

Supplementary Figure 5 | Static absorption spectrum of phenoxide. UV-visible absorption spectra of 0.2-M sodium phenoxide in water.

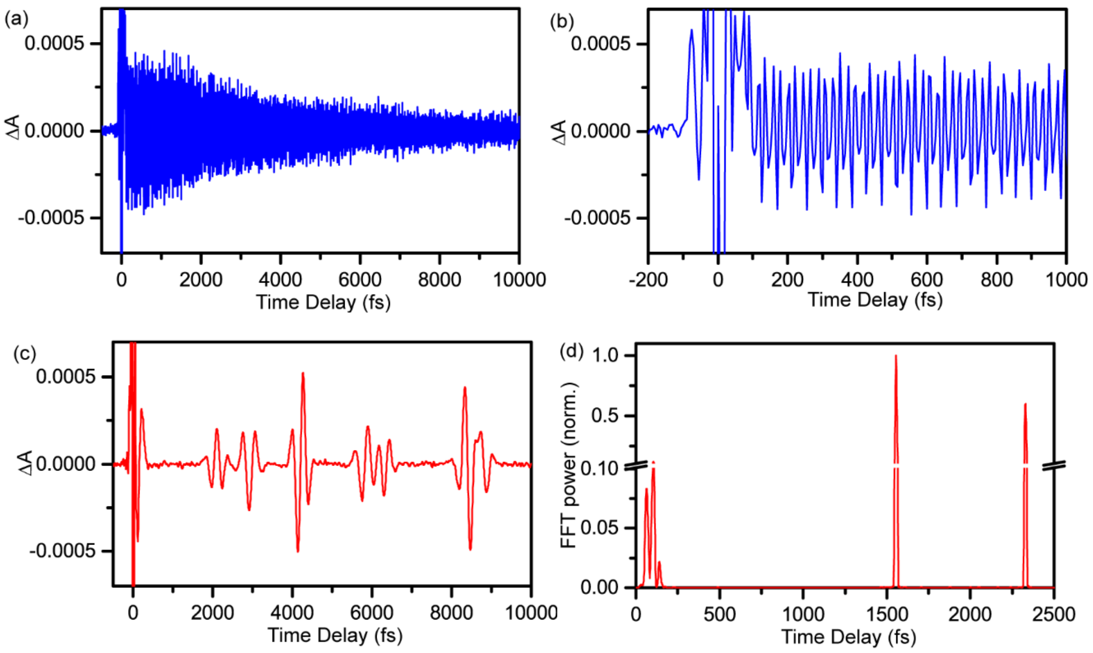

Supplementary Figure 6 | Rovibrational wave packet dynamics of air. (a) Time-resolved differential absorption signal at 400 nm recorded in air, in the absence of the liquid microjet target. (b) Detail of differential absorption time trace, plotted from -200 to 1000-fs time delay, clearly showing modulations as a function of time. (c) Low-pass Fourier-filtered time trace of the ΔA time trace shown in (a), revealing slow modulations due to rotational wave packet revivals. (d) FFT power spectrum of the ΔA time trace. Note the break after 0.1 in the vertical axis.

Supplementary Table 1. Optimized Cartesian coordinates of the isolated phenoxide ion.

	Atom	X (Å)	Y (Å)	Z (Å)
1	C	-1.07779	-2E-06	-1E-06
2	C	-0.28749	1.21183	0.000003
3	C	1.100174	1.200007	-1E-06
4	C	1.82787	-1E-06	0.000002
5	C	1.100175	-1.20001	-2E-06
6	C	-0.28749	-1.21183	0.000003
7	O	-2.34719	0.000001	-2E-06
8	H	-0.83139	2.15339	0.000001
9	H	1.637119	2.148505	0
10	H	2.913339	0.000001	-1.3E-05
11	H	1.637117	-2.1485	0.000001
12	H	-0.83139	-2.15339	0.000001

Supplementary Table 2. Optimized Cartesian coordinates of the isolated phenoxyl radical.

	Atom	X (Å)	Y (Å)	Z (Å)
1	C	-1.04711	0	-8E-06
2	C	-0.28974	1.238907	-6E-06
3	C	1.085525	1.223844	0.000001
4	C	1.782396	0	0.000005
5	C	1.085525	-1.22384	0.000001
6	C	-0.28974	-1.23891	-6E-06
7	O	-2.30019	0	0.000011
8	H	-0.85531	2.163307	-0.00001
9	H	1.642283	2.154395	0.000001
10	H	2.866395	-1E-06	0.000013
11	H	1.642282	-2.1544	0.000001
12	H	-0.85531	-2.16331	-0.00001

Supplementary Table 3. Optimized Cartesian coordinates of the phenoxide ion in aqueous solution.

	Atom	X (Å)	Y (Å)	Z (Å)
1	C	0.34464	0.02777	-0.00253
2	C	1.0844	-1.18034	-0.03869
3	C	2.47722	-1.17722	-0.04458
4	C	3.19322	0.023	-0.01468
5	C	2.48116	1.22523	0.02192
6	C	1.08818	1.23359	0.02786
7	O	-0.98131	0.02552	0.00384
8	H	0.53908	-2.11871	-0.06093
9	H	3.01008	-2.12306	-0.07246
10	H	4.27723	0.02137	-0.01924
11	H	3.01726	2.16936	0.04643
12	H	0.5486	2.17477	0.05763
13	O	-2.19161	-1.16178	2.11189
14	O	-2.2383	-1.42672	-1.89854
15	O	-2.46002	2.26872	-0.09381
16	H	-1.73426	-0.72645	1.34754
17	H	-2.29814	-0.46631	2.76853
18	H	-1.76155	-0.90015	-1.20577
19	H	-2.52431	-0.77999	-2.55134
20	H	-1.86797	1.46921	-0.07711
21	H	-2.24901	2.72188	-0.91619

Supplementary Table 4. Optimized Cartesian coordinates of the phenoxyl radical in aqueous solution.

	Atom	X (Å)	Y (Å)	Z (Å)
1	C	0.28512	-0.03826	0.00674
2	C	0.96629	-1.31624	-0.01103
3	C	2.33758	-1.37057	-0.00799
4	C	3.0949	-0.18	0.01255
5	C	2.46087	1.08059	0.03062
6	C	1.09111	1.1647	0.02798
7	O	-0.98131	0.02552	0.00384
8	H	0.35885	-2.21307	-0.02593
9	H	2.8464	-2.32677	-0.02115
10	H	4.1768	-0.23444	0.01471
11	H	3.06348	1.98055	0.04675
12	H	0.57786	2.11848	0.04216
13	O	-2.63205	-1.33746	2.02316
14	O	-2.57709	-1.4897	-1.94574
15	O	-2.55687	2.42724	-0.02065
16	H	-2.0865	-0.91291	1.34238
17	H	-2.79228	-0.64668	2.67514
18	H	-2.0761	-1.02674	-1.2553
19	H	-3.49812	-1.26796	-1.77142
20	H	-1.99783	1.62998	-0.04605
21	H	-2.62495	2.71221	-0.93801

Supplementary Table 5. Partially optimized Cartesian coordinates of the phenoxyl radical in aqueous solution, with the microhydrating water molecules frozen in the same geometry as in phenoxide.

	Atom	X (Å)	Y (Å)	Z (Å)
1	C	0.29621	0.02797	-0.00429
2	C	1.03324	-1.21556	-0.03359
3	C	2.40462	-1.20357	-0.0383
4	C	3.10264	0.0235	-0.01486
5	C	2.4081	1.25241	0.01523
6	C	1.03669	1.26937	0.02024
7	O	-0.98131	0.02552	0.00384
8	H	0.4696	-2.14021	-0.0497
9	H	2.95944	-2.13336	-0.05967
10	H	4.18582	0.02208	-0.0188
11	H	2.9659	2.18045	0.03519
12	H	0.47837	2.19688	0.04618
13	O	-2.21499	-0.94347	2.20781
14	O	-2.24926	-1.58335	-1.76022
15	O	-2.43082	2.26774	-0.31153
16	H	-1.7491	-0.58754	1.40832
17	H	-2.3152	-0.18812	2.79572
18	H	-1.76856	-0.99997	-1.11751
19	H	-2.52441	-0.99732	-2.4723
20	H	-1.84911	1.46607	-0.21682
21	H	-2.2108	2.63899	-1.17173

Supplementary Table 6. Results of DFT calculations, showing the bond lengths of the phenoxide ion and the phenoxyl radical, and the difference bond lengths, in both gas phase and aqueous solution.

Bond	Bond Length / pm		Diff. / pm	Bond Length / pm		Diff. / pm
	PhO^{-} (g)	PhO· (g)		PhO^{-} (aq)	PhO· (aq)*	
C1 – C2	144.7	145.2	-0.5	141.7	144.6	-2.9
C2 – C3	138.8	137.5	1.3	139.3	137.2	2.1
C3 – C4	140.3	140.8	-0.5	139.8	141.2	-1.4
C4 – C5	140.3	140.8	-0.5	139.8	141.2	-1.4
C5 – C6	138.8	137.5	1.3	139.3	137.1	2.2
C6 – C1	144.7	145.2	-0.5	141.7	144.6	-2.9
C1 – O7	126.9	125.3	1.6	132.6	127.8	4.8

* The water molecules of microhydration and the H_2O•••O(phenoxide) distances are not relaxed upon photodetachment.

Supplementary Note 1: Photon-order measurement for the strong-field photodetachment of phenoxide

Power dependence measurements were performed to determine the photon order of the strong-field photodetachment process. For an N-photon process, the differential absorption signal ΔA scales with power P as $\Delta A \propto P^N$. The photon-order measurements were performed by using a visible probe pulse instead of a 400-nm probe pulse. The visible probe pulse, a replica of the strong-field photodetachment pulse, measures the absorption of the hydrated electron at a pump-probe time delay of 500 fs and a wavelength of 680 nm. Since the hydrated electron is a byproduct of phenoxide photodetachment and water photoionization, the hydrated electron absorption signal is proportional to the extent of photodetachment and photoionization.

To correctly determine N, one must consider the simultaneous electron ejection from both the phenoxide solute and the water solvent, even though ionization of the solute is greatly favored due to its significantly lower vertical ionization potential ($\mathrm{IP}_{\mathrm{PhO}^-} = 7.1$ eV vs. $\mathrm{IP}_{\mathrm{H_2O}} = 11.16$ eV). In our experiments, we record ΔA vs. P for two different samples: (1) aqueous phenoxide, and (2) pure water. The difference between the two sets of data originates from ionization from phenoxide itself. The log-log plot of the difference ΔA ($\Delta\Delta A$) vs. P is shown in Figure S3. In the regime before saturation, the data can be fit to give a slope of 4.1 ± 0.1, which in turn suggests photodetachment via a four-photon process.

Supplementary Note 2: Rovibrational wave packet dynamics of N_2 and O_2

To verify that the frequencies at 1555 and 2330 cm^{-1} (see Table 1 of the main text) originate from vibrational wave packet motion of O_2 and N_2, respectively, we performed pump-probe experiments in the absence of the liquid microjet target, i.e., in air. The experimental conditions were identical to those employed in the time-resolved spectroscopy of photodetached phenoxide. The resultant ΔA time trace recorded at 400 nm is shown in Supplementary Figure 6a; the corresponding FFT power spectrum is shown in Supplementary Figure 6b. In addition to the vibrational frequencies, a band of low frequencies are also observed due to rotational wave packet dynamics (Supplementary Figures 6c & 6d). These rovibrational wave packet dynamics most likely arise from impulsive stimulated Raman scattering.

Supplementary Note 3: Sequence bands in the gas-phase photodetachment spectrum of phenoxide

The Franck-Condon progression in the gas-phase photodetachment spectrum (ref. 27 in the main text) comprises two series of peaks: one for 11_0^n and another one for $14_1^1 11_0^n$, where ν_{11} and ν_{14} correspond to the phenoxyl vibrational modes with frequencies 519 and 369 cm^{-1}, respectively. The latter progression corresponds to a series of sequence bands involving the ν_{14} mode trailing the ν_{11} progression. These bands are observed because the vibrational temperature of the phenoxide precursor, estimated to be ~300 K, gives rise to a finite population of the ν_{14} mode in the phenoxide precursor (ν_{14} = 420 cm^{-1}). Note that there is no change in the vibrational quantum number of the ν_{14} mode accompanying photodetachment. Peaks that appear in the photodetachment spectra, after subtracting the electron affinity offset, correspond to differences between the vibrational energy levels of the phenoxide anion and the phenoxyl radical. This explains the –51 cm^{-1} downshift in the position of the sequence bands relative to the ν_{11} progression. In our measurements by femtosecond wave packet spectroscopy, the oscillation frequencies that are observed correspond to differences between the vibrational energy levels of the phenoxyl radical itself, i.e., the wave packet motion involves only the phenoxyl radical. As such, we do not expect to observe vibrational frequencies that are associated with the sequence bands that are identified in the gas-phase study.
